# Common Mental health issues among non-refugee migrants in Australia: a scoping review

**DOI:** 10.1007/s00127-025-02850-2

**Published:** 2025-02-19

**Authors:** Pritimoy Das, Colette Browning, Muhammad Aziz Rahman

**Affiliations:** 1 Institute of Health and Wellbeing, Federation University Australia, Ballarat, VIC 3350 Australia; 2 Health Innovation and Transformation Centre, Federation University Australia, Victoria, Australia; 3 Research School of Population Health, Australian National University, Canberra, ACT Australia; 4 Institute of Health and Wellbeing, Federation University Australia, Canberra, VIC 3806 Australia; 5 Faculty of Public Health, Universitas Airlangga, Surabaya, Indonesia

**Keywords:** Australia, Migrant, Psychological distress, Prevalence, Risk-factors

## Abstract

**Purpose:**

Mental health issues were the fourth leading cause of disease burden in Australia in 2022. About 30% of Australia’s population are migrants, whose mental health is poorly understood. We aimed to report the prevalence and risk factors of common mental health issues among non-refugee migrants in Australia.

**Methods:**

We reviewed studies published between 2000 and 2024 on mental health issues amongst migrants in Australia following the Arksey and O’Malley’s methodological framework and PRISMA-ScR guidelines.

**Results:**

Out of 3122 titles retrieved on mental health issues among migrants in Australia, 30 papers were selected. Migrants from Greece reported the highest prevalence (43.1%) of anxiety disorders than Australian-born (15.8%). The highest prevalence of psychological distress and depression were found amongst migrants from Lebanon (33%) and China (19%), respectively. Migrants from North-Africa, the Middle East, Italy, Greece, and Europe experienced a higher prevalence of psychological distress (18.2–21.9%) than Australian-born (12.4%). Prevalence of depression was higher among migrants from non-English-speaking backgrounds (19.7% vs. 10%), Sub-Saharan Africa (18.8% vs. 9.3%), Italy (18% vs. 10%), Greece (17.1% vs. 4.1%), and China (10% vs. 3%), compared to Australian-born people, respectively. The way that risk factors were reported differed across studies. Anxiety disorders were associated with higher stress (p < 0.05), unemployment (OR 1.8, 95%CI:1.4–2.4), female gender (OR 2.13, 95%CI:1.64–2.76) unmarried status (p < 0.01) and poor physical health status (OR 7.35, 95%CI:3.86–14.01). Psychological distress was associated with being a single woman (OR 6.54, 95%CI:1.18–35.3), holding a temporary visa (p < 0.01), being economically inactive (p < 0.01) and having rare contact with friends (AOR 2.083, p < 0.001). Depression was associated with migrants who were never married (OR 4.11, 95%CI:1.59–10.65), younger or older (p < 0.001), female (OR 2.3, 95%CI:1.9–2.7), from non-English speaking countries (OR 2.41, 95%CI:1.14–5.10) and reported poor physical health (OR 3.55, 95%CI:1.60–7.88).

**Conclusion:**

The high prevalence of mental health issues among non-refugee migrants necessitates revisiting strategies to tailor interventions appropriate for their mental health needs.

**Supplementary Information:**

The online version contains supplementary material available at 10.1007/s00127-025-02850-2.

## Introduction

International migration has greatly increased over the past 50 years. About 281 million people were residing in countries other than their countries of birth in 2020, which was over three times the estimated numbers in 1970 [1]. The demographic changes in the Australian population have been significantly influenced by international migration. Before British migrants came to Australia in 1788, only Indigenous people lived in Australia [2]. In 1966, the Holt government reformed immigration policies towards eliminating the long-held ‘White Australia’ policy. These modifications led to the formation of a multicultural Australia [3]. New migrants from a variety of ethnic and cultural backgrounds have arrived in recent years compared to the earlier White and European settlers [4]. Australia has people from nearly 200 countries who represent more than 300 ethnic ancestries in 2022 estimate [5]. This migrants constitute 29.8 percent of the Australian population and comprise over 7.6 million people [6]. England (12.8%), India (9.5%), China (8.5%), New Zealand (7.4%) and the Philippines (4.1%) are the top five contributing countries sending migrants in Australia [6]. Refugee and humanitarian entrants are also welcomed to Australia in addition to the regular (voluntary) migration.

Migrants can enter Australia as either permanent or temporary residents. Australia had 1,614,000 temporary residents in 2021; the majority of the temporary residents were either working holidaymakers, skilled workers, or students [7]. Permanent migrants enter Australia via one of two distinct programs—the Migration Program for skilled and family migrants or the Humanitarian Program for refugees and asylum seekers. Report shows that until 2021, there were 3.0 million permanent migrants in Australia who arrived since 2000; of these, 59% entered the country through the skilled visa stream, 32% through the family visa stream, and the remainder of the population entered through the humanitarian visa stream [8]. According to the United Nations High Commissioner for Refugees (UNHCR), Australia now hosts almost 60,000 refugees and 80,000 asylum seekers (people who have been forced to flee their homes and have crossed an international border to find safety in another country), most are from the Middle East or Asia [9]. For the purpose of this paper, we described “non-refugee immigrants” as all migrants excluding those who arrived through this special humanitarian stream.

Pre-migration and migration factors may affect migrants’ mental health (e.g., poor living conditions in their home country, discrimination, etc.). Many studies reported that refugee immigrants often experience significant trauma before migration, such as war or persecution, leading to higher rates of PTSD, anxiety, and depression compared to non-refugee immigrants. They tend to have less control over their migration process, which can exacerbate feelings of loss and powerlessness, and face greater barriers to healthcare access. In contrast, non-refugee immigrants typically migrate for economic or educational reasons, have more control over their migration, and usually migrate for a better future. However, although non-refugee migrants constitute the majority of the overall migrant population in Australia, their mental health issues were less focused.

Restarting a new life in a new country with potentially different social and cultural norms and values can have enormous impacts on the health and wellbeing of migrants, their families, and communities. Every person who leaves his/her own country of birth goes through emotional challenges. They are propelled by the hope of experiencing a better life. Dealing with everyday stressors after migration poses a higher risk for psychological issues, depression and comorbidity [10]. Factors implicated in this complex adaptation process include acculturation, dietary transitions, adoption of different lifestyles, one’s race/ethnicity, gender, socioeconomic status, and relationships to the state and its institutions including health services. This complexity adds further stress, anxiety, and depression [11, 12].

Australia, Canada, Europe, and the United States of America (USA) are among the top developed countries popular destinations for migration. The prevalence of psychological distress and depression amongst migrants was explored in different migrant-hosting countries. A large study conducted in the USA revealed that- among 131,669 USA-born and 26,155 non–USA-born participants, the prevalence of psychological distress was 3.0% and 2.2%, respectively [13]. Other studies reported that the prevalence of depression in the USA was found to be 13.2% amongst migrants from South Korea [14], 11% from Mexico [15], and 5%-7.8% from China, Vietnam, Philippines and other Asian countries [16, 17]. Seven European countries reported significantly higher levels of depression among migrants than non-migrant populations [18].

Mental disorders ranked as the fourth highest disease group contributing to Australia’s total burden of disease in 2019, [19]. One in five Australians (4.8 million) reported having a mental or behavioural problem [20]. Of these, depression, anxiety disorders and psychological distress were most common among migrants in Australia [20, 21, 21]. Research shows that the likelihood of developing a mental illness is relatively high among migrants [22]. Apart from this, researchers found disparities in healthcare-seeking behaviours and healthcare service access among different migrants compared to the Australian-born host population. Up to 91% of Indian migrants in Australia with identifiable mental health issues did not seek any mental health consultation [23]. The major barriers to accessing healthcare include language barriers during health communication [24], social stigma and self-stigma [25], migrants’ socioeconomic disadvantage [26] and inadequate social support due to a lack of culturally appropriate services [27].

Migrant health protection is a significant public health investment since it benefits both migrants and the Australian-born population [28]. Although a few attempts were taken to evaluate migrants’ mental health, most studies were conducted targeting a specific ethnic origin or country of birth [23, 29–31], or limited to specific age or sex groups [32–34], or were derived from a secondary data source limited by low (18%) response rate [27, 35]. However, very few studies assessed risk factors associated with mental health issues or compared their findings with the corresponding host population, which could identify gaps and help to deal with social inequalities. Considering the importance of providing optimal care for migrants, defining priorities and designing targeted health promotion programs and appropriate interventions for the prevention and control of mental health issues among migrants, it is necessary to gain an overview of migrants’ mental health issues in Australia. There is a clear need to review and collate research to answer the research question: what is known about the common mental health issues among non-refugee migrants in Australia? Therefore, we conducted a scoping review to better understand the prevalence and risk factors of common mental health issues such as anxiety, depression, and psychological distress amongst non-refugee migrants in Australia and compared them with non-migrants.

## Materials and methods

### Study design

We conducted a scoping review based on the Arksey and O’Malley’s methodological framework [36]. This review was further guided by Levac et al. [37] and the Joanna Briggs Institute [38]. We also followed the PRISMA-ScR (Preferred Reporting Items for Systematic Reviews and Meta-Analyses for Scoping Review) checklist [39] to ensure the authenticity, as well as standardization of the scoping review process.

Arksey and O’Malley’s methodological framework for conducting a scoping review provides a structured approach to identifying, selecting, and analyzing relevant research literature on a particular topic. It involves six stages: identifying the research question; identifying sources of data; selecting studies; data charting; summarizing and collating results; and reporting the review. In addition, we assessed the included studies for methodological quality as recommended by Levac et al. (2010) and the Joanna Briggs Institute.

### Study population

The study population for this scoping review was migrants in Australia. The Australian Bureau of Statistics defines a migrant as “a person who was born overseas whose usual residence is Australia” [40]. For this study, we also considered this definition. A non-migrant is someone who was born in Australia, even if his/her parents are migrants. All migrants, irrespective of their age, sex, and country of birth, who were recruited from the general population or attended any healthcare facility, were considered for this scoping review.

### Study period

Articles published between January 2000 and August 2024 were considered in this scoping review. This timeframe has been chosen because there have been substantial advancements in non-communicable diseases research in the last two decades, such as the establishment of different prospective cohorts [41] and changing patterns of global migration in recent years [42].

### Study selection

We developed a search strategy in consultation with the Federation University Australia’s senior librarian who has expertise in systematic literature search. The following conceptual areas were searched using a combination of Medical Subject Headings, free text, and indexing terms: “Migrant”, “Australia” and “Mental health issues” (Box 1). We selected four online databases for the literature search relevant to our study area: MEDLINE, CINAHL, Scopus and Web of Science. We also read the references of the review papers to find any additional papers that were not retrieved from the online database search.

## Box 1. Keywords and index terms to construct search strategy used for the scoping review

**Table:** 

Search query	Focus	Keywords
1	Migrants	“Transients and Migrants” OR Migrant* OR Immigra* OR migration OR ethnicity OR multicultural
2	Common mental health issues	“Mental Disorders” OR Psychiatric OR Depression OR “anxiety disorder” OR “psychological distress” OR “mental health”
3	Australia	Australia* OR Victoria* OR “New South Wales” OR “Northern Territory” OR Queensland OR “South Australia” OR Tasmania OR Melbourne OR Sydney OR Adelaide OR Perth OR “Western Australia”
4	Final search	1 AND 2 AND 3

NB. Boolean Operators (AND, OR) were used as conjunctions to combine or exclude keywords in a search to retrieve more focused results

### Inclusion/exclusion criteria

We included peer-reviewed original research articles reporting prevalence and/or factors associated with common mental health issues (anxiety disorders, depression and psychological distress) amongst migrants in Australia with/without comparison with non-migrants. Papers reporting the mean number of anxiety, depression or psychological distress symptoms or symptom scales among migrants and comparing them with non-migrants with or without reporting prevalence/risk factors were also included. This review excluded studies that only discussed experiences or a case report about a single migrant, studies using qualitative design, studies about ‘First Nation Peoples’ in Australia and systematic reviews. Articles on severe forms of mental illness such as suicide, psychosis and substance use disorders related papers were not considered ‘common mental health issues’, thus excluded. As refugees and asylum seekers who were 3.2% of the migration program in Australia [43] bear a special status and pass through special immigration policies and international protection laws than the regular migrants, we excluded papers on refugees and asylum seekers.

### Databases

MEDLINE and CINAHL were searched using the EBSCOhost interface, and Web of Science and Scopus were searched using their web interface.

### Search strategy and publication selection

The first author (PD) conducted the online search following the search strategy mentioned earlier. The titles and abstracts of all the studies derived from the four databases were imported into Covidence^®^ to manage the review process [44]. At first, Covidence^®^ automatically removed the duplicates and generated a PRISMA flow diagram. After that, the remaining studies were available for screening. PD conducted the initial screening of the titles and abstracts in the Covidence^®^. A second researcher assessed 10% of the title and abstracts to ensure the quality of the screening. The full texts of the screened articles were then reviewed in detail by the first author (PD) and the second review was done by the other two authors (CB and MAR) for eligibility of the final inclusion. Any specific reasons for the exclusion of full-text studies were recorded. The conflicts were resolved by having a discussion among the co-authors. We refined the selection of articles during these discussions until we reached an agreement.

### Data extraction and synthesis

A standardized extraction form in Excel was developed using some of the included full-text papers as a guide to identify necessary variables and study characteristics to assist in article selection: author(s), year of publication, study period, source of data, research questions/objectives, study design and setting, study population and sample size, potential factors influencing the mental health issues, prevalence, and other significant findings. Then data were extracted from selected papers by the first author into the Excel sheet and reviewed by all co-authors. A narrative synthesis of the findings was conducted and was reported in this paper.

### Ethics and dissemination of review findings

This study does not require ethical approval since the scoping review methodology aims at synthesizing information from secondary data sources (publications).

### Quality assessment

The quality of the selected studies was assessed using the adapted Newcastle Ottawa Scale (Appendix 1) used for systematic review [45]. It evaluates studies based on three main criteria: selection of study groups, comparability of groups, and the ascertainment of either the exposure or outcome. Each criterion is scored with stars to indicate methodological rigor, helping to ensure that results are not biased by flaws in study design or execution. A study with a higher score is generally considered to be of higher quality.

## Results

### Characteristics of the studies

**Fig. 1:**
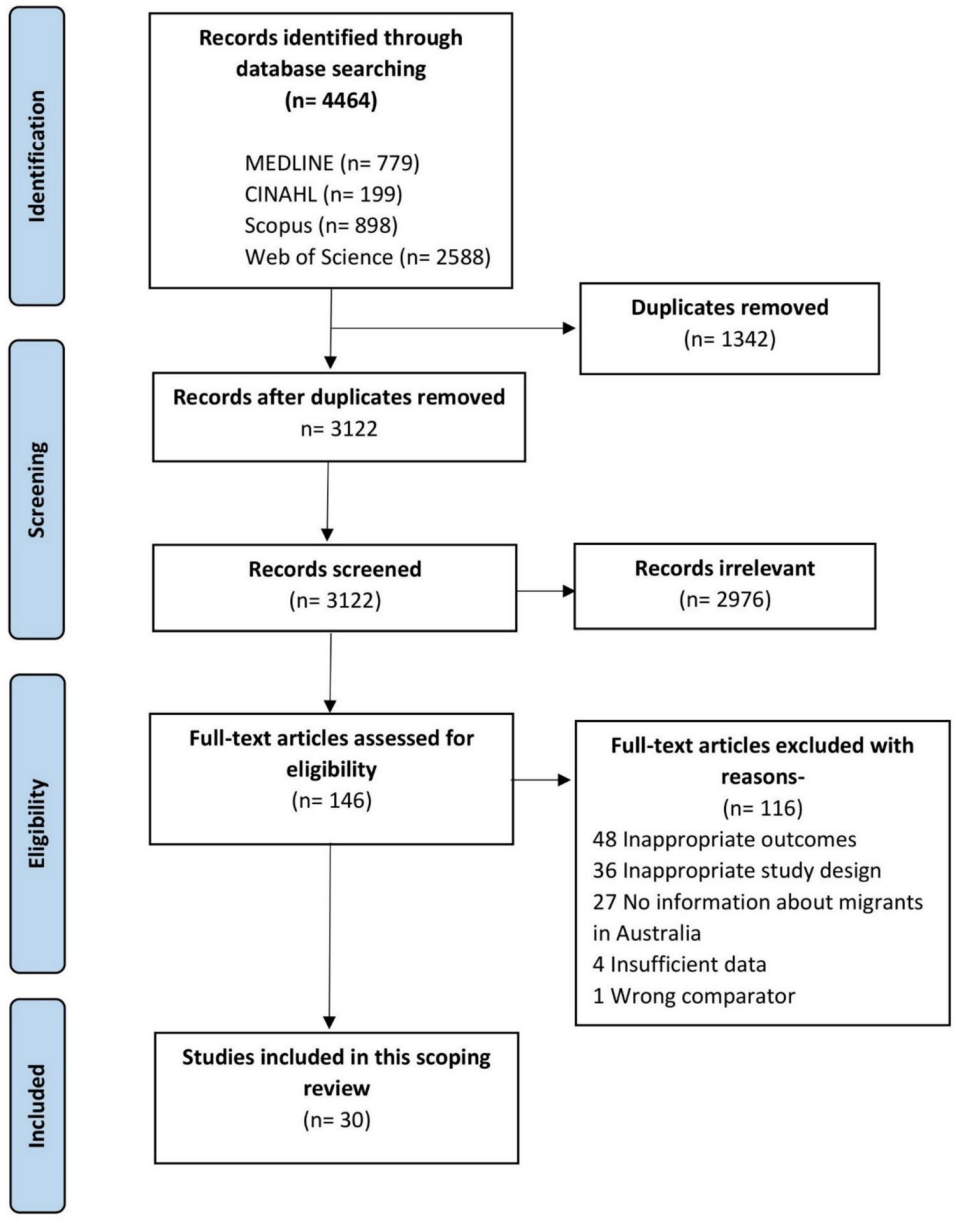
Study selection process for the scoping review on mental health issues among non-refugee migrants in Australia

Out of 3,122 unique articles revealed through the database search after duplicates removal, 30 papers were finally selected for this scoping review (Table 1 and Fig. 1). Most were cross-sectional studies (n = 27), followed by a few longitudinal studies (n = 3). Most of the studies (n = 26) used face-to-face interviews/telephone/postal surveys, while four applied online survey methods. One in four studies (n = 6) analyzed secondary data (e.g.- from the 45 and Up study) or data obtained from the Australian Government (e.g.- the National Survey of Mental Health and Wellbeing conducted by the Australian Bureau of Statistics). The studies were conducted in various states of Australia: ten in New South Wales, seven in Victoria, four in Queensland, one in Northern Territory and one in South Australia. Seven studies involved respondents from all Australian states and territories. None of our included studies involved respondents specifically from rural Australia. Sample sizes and study population varied across studies from 46 Filipino mothers in one study to 228,039 migrants from multiple countries in another study [27, 33]. Although most studies included both men and women, two studies included only women [33, 46]. We found that, there were methodological differences across studies, as well as heterogenicity of assessment tools and instruments. The tool ‘Kessler Psychological Distress Scale (K-10)’ developed by Kessler et al. [47] was used most, while other studies used different instruments (Appendix 2) for assessing mental health issues.

**Table 1:** Description of included studies for the scoping review- prevalence and risk factors of mental health issues among migrants in Australia (2000–2024)

Study	Study design, state/ city	Data source &/or study period	Sample size/participants /age range	Focus	Comment/critics
Comino (2001) [48]	Cross sectional survey New South Wales, Sydney	1993	n = 4753 18 to 90 years old participants with no major psychiatric disorders presented at 117 GP clinics in Sydney AB 3040 ESB 485 European 640 Asian 299 Other NESB 289	Anxiety, depression	No risk factor analysis. This study assessed prevalence and compared findings from three methods of detecting anxiety/depression
Thompson (2002) [46]	2 surveys, 1 year apart. Home or phone interview Queensland, Brisbane	1996/1997. Follow-up in 1997/1998	n = 346 Filipino women, (71% response rate at f/up) Mean age 42.3 years	Depression	1. The contribution of life events and satisfaction with life in Australia was captured for the past 12 months only, so events responsible for mental distress beyond this time was unknown 2. Reported in terms of above-threshold GHQ-28, instead of “Depression”
Kiropoulos (2004) [29]	Cross-sectional Victoria, Melbourne	Sixteen Greek community social clubs. Study period not found	146 were Greek and 146 were AA Included only those aged ≥ 50 years	Anxiety, depression	1. Respondents were selected from among those in contact with social clubs and were self-selected to the study and thus may not be representative of older people in Melbourne
Alati (2004) [33]	Longitudinal Queensland, Brisbane	The data was taken from the Mater-University Study of Pregnancy (MUSP): carried out in Brisbane, Australia since 1981	Filipino mother n = 46 At their first clinic visit, there were 87 Filipino-born mothers who agreed to be interviewed. Followed up at five years and 14 years after the birth of the child	Anxiety, depression	1. May be under reported. Filipinos conceal mental problems, because of the stigma attached to ‘madness’ in Filipino society 2. Possible bias resulting from loss at F/U. There are no indication that Filipino-born mothers lost at F/U had poorer mental health compared with those who remained in the study 3. No risk factor analysis
Khawaja (2007) [49]	Cross-sectional Queensland, Brisbane	Mosque, other Muslim cultural, social, and student organizations, questionnaire fill up, mail return	280 Muslims. mean age was 33.77 years (SD: 12.31 years). The participants originated from 43 different countries and were categorized into six geographical regions: Asia (25%), Middle East (24%), Africa (20%), Fiji (12%), Australia (11%), and Europe (6.4%)	Depression	1. The response rate was low 20% 2. There is a possibility that many others who took the questionnaires could either not fully understand the scales due to limited English and Arabic language 3. Not analyzed by COB among Muslims
Chou (2007) [50]	Longitudinal Australia	List of immigrants were from the first and second waves of the Longitudinal Survey of Immigrants to Australia (LSIA), Interview: 2000–2001	n = 431, 359 f/u after 1 year Random, all selected migrants were aged 50 + years Asian countries 44.7%, Western and developed countries 21.5%, Other countries 33.8%	Psychological distress	1. More than half of subjects were interviewed with the assistance of interpreters, and it is difficult to determine the quality of interpretation 2. The present study did not include measures of acculturation, measure of social support, functional impairment, and stress or critical life events 3. Only 1 year period is not a good for a longitudinal study. a longer period, say 5 years, are needed to further understand the development of mental health problem among immigrants in Australia
Steel (2009) [51]	Comparative analysis of three multistage population surveys New South Wales	The Australian Vietnamese survey was conducted between June 1999 and May 2000. The Australian Bureau of Statistics survey was conducted between May 1997 and August 1997	Vietnamese immigrants residing in New South Wales, Australia (n = 1161), and Australian-born host population (n = 7961) Age 18 + years	Mental disorders	1. There was a difference in the timing of the investigations, with Mekong Delta Vietnamese being studied more recently 2. Unable to sample very low-density suburbs, possibly excluding more acculturated Vietnamese
Tang (2009) [52]	Cross sectional survey, patients attending a general practice in southwestern Sydney (New South Wales)	2005	A total of 161 Chinese patients, the mean age of respondents was 47.6 (SD: 13.7) years of age; 70% were female	Depression	A small survey conducted in a single suburban general practice where the majority of patients are of immigrant Chinese background. The results, therefore, may not be applicable to the wider Chinese population
Stanaway (2010) [32]	Cross-sectional, New South Wales, Sydney	The Concord Health and Ageing in Men Project (CHAMP) 2005–2007	335 Italian-born and 849 Australian-born host population, men aged 70 years and over	Depression	1. Did not include women in this study 2. use of the English version in this subgroup may have underestimated depressive symptoms
Sharma (2012) [34]	Secondary data analysis Australia	National Survey of Mental Health and Wellbeing, 2007	n = 6529, ESC 1032, NESC 1278 Age 16–85 years	Psychological distress	The survey excluded those who could speak no English and only 60% of the target sample were interviewed; almost nothing is known about the remaining 40% of the adult population
Maheshwari (2012) [23]	Cross-sectional New South Wales, Sydney	May and July 2009	71 Indian family groups. Above 15 years of age and with a birth or ancestral link with India. Included international students but excluded other short-term visa-categories such as those on tourist visit visas	Psychological distress	1. Small sample size and restricted geographical coverage limit the generalizability of the findings 2. ‘Indian-Australians’ as a single group without any distinction based on languages, religions, dietary practices, migration histories or socio-demographic background 3. The recruitment strategy may have excluded sections of the community who do not access the spice shop
Kiropoulos (2012) [53]	Cross-sectional Victoria, Melbourne	Face-to-face interview 2009–2011	Greek-born n = 61 Anglo-Australian n = 62 In two coronary care units in major hospitals in Melbourne	Anxiety, depression	1. The sample size for the groups examined in this study was small, participants were recruited from two coronary care units of the same hospitals and may not have been a representative sample of the populations examined
Feng (2013) [35]	Cross-sectional, secondary data analysis New South Wales	45 and Up study 2006–2009	19 ethnic groups, 45 years and over	Psychological distress	1. 45 and Up Study was sampled from the Medicare Australia database which mainly includes Australian citizens and migrants on permanent residency visas. Only some migrants on temporary visas are included in this scheme and this is likely to mean that some ethnic minorities were not represented 2. Response rate was only 18% 3. No risk factor analysis but COB
Liddell (2013) [54]	Cross sectional study New South Wales	Vietnamese-immigrant survey: June 1999—May 2000 AB: National survey of mental health (ABS), 1997	Vietnamese migrant 1161, AB- 7961 Adults over 18 years	Anxiety, depression	It was not possible to assess the specific impact of culture and migration experiences since these factors were only relevant to one group, the Vietnamese migrants
Straiton (2014) [21]	Cross-sectional secondary data analysis South Australia, Adelaide	The North West Adelaide Health Study (NWAHS) cohort 2004–2006. Telephone interview	Only adults who were under 65 years at time of data collection (n = 2,605) Australian-born host population 1,895, Foreign-born (English speaking background) 417, Foreign-born (non-English speaking background) 292	Depression	1. Lacked the power to investigate differences by country or world region. Those who may experience the greatest barriers to care, and the poorest mental health may have been excluded 2. This study also excluded people without a telephone or who were not listed in the white pages 3. The attrition rate of around 10% from stage 1 of recruitment to stage 2 of the study may be a concern since those with poorer mental health may have been more likely to drop out
Meng (2014) [55]	Cross-sectional New South Wales, Sydney	During a 7-week period, when Chinese patients attended the Diabetes Centre of Royal Prince Alfred Hospital for a routine visit in 2012	100 Chinese people with diabetes born outside of Australia and who have resided in Australia for at least one year	Depression	Sample size was relatively small; all participants were from a single specialized outpatient clinic 2. The effects of chronic conditions on the observed depression were not analyzed and should be tested in future studies
Ferdinand (2015) [56]	Cross-sectional Victoria	The CALD Experiences of Racism surveys conducted in 2010	N = 1139 aged 18 years and older and lived within Rural Council 1 (n = 298), Metropolitan Council 1 (n = 335), Metropolitan Council 2 (n = 226) or Rural Council 2 (n = 280) for at least one year	Psychological distress	The data is cross-sectional, so there is the potential for reverse causation; that is, people above the threshold for high or very high psychological distress may be more exposed to racism
Khawaja (2016) [57]	Cross-sectional QLD, Brisbane	Online link and hard copy survey. Date not identified	Taiwanese migrants (N = 271), mean age was 33.7 years (range 18–71 years; SD = 12.86)	Psychological distress	Participants were from one small area; the sample may not adequately represent the overall Taiwanese migrants settled in Australia
Lin (2016) [30]	Cross-sectional Victoria, Melbourne	Face to face interview. Period not found	Chinese 59, Australian-born host population 60 Age > 65 years, > 3 years of residence	Anxiety and depression	1. Small sample size, convenient sampling- poor generalizability of results 2. Chinese participants were recruited form community groups and engagement in social activities- > less depression No separate analysis for predictors among the 2 groups
Hu (2016) [58]	Online survey Australia	Online health survey from July to October 2013	414 Chinese migrants. Chinese migrants who lived in Australia longer than 3 months were recruited through several Chinese social websites to participate in an online health survey	Psychological distress	1. Convenience sampling method and the sample population was overly represented by the participants from mainland China (92%) 2. Survey is likely to miss out those older Chinese migrants who may utilise the internet less
Liddell (2016) [59]	Secondary data analysis Australia	National Survey of Mental Health and Wellbeing (NSMHWB) conducted by the Australian Bureau of Statistics (ABS) in 2007	n = 8,841, persons aged 16–85 years	Anxiety and depression, Common mental disorders	1. Interview was conducted only in English 2. The data on specific countries-of-origin of respondents was not available from the ABS dataset accessed by the authors 3. contribution of specific cultural backgrounds ignored 4. Future studies will need to expand beyond English-speakers and single immigrant groups
Hosseini (2017) [60]	Cross-sectional Australia	Online questionnaire- June 2012 and February 2013. Online questionnaire was conducted through 17 Iranian non-government organizations (NGOs) across Australia	182 Iranian immigrants aged 18 years or older, living in Australia and able to communicate in Farsi (Persian) or English	Migration experience, resilience and depression	1. Authors reported Higher level of depression among participants, but prevalence data was not mentioned 2. The use of convenience sampling from the Iranian NGO community limits the generalizability of results to the population of Iranian immigrants in Australia because of sampling bias
Nahidi (2018) [61]	Cross-sectional New South Wales, Sydney	Iranian international students enrolled in different disciplines at UNSW during the second semester of 2012 or the first semester of 2013	n = 180, age 18–40 years, male 61%	Psychological distress	The sample was recruited from only one Australian University. There is potential for self-selection and social desirability biases, thus findings should be taken cautiously
Dow (2018) [62]	Cross-sectional Victoria, Melbourne	May and June 2014 A convenience sampling approach was used	87 older Chinese migrants, ranged in age from 60 to 92 years. 66% Female	Depression Anxiety	Based on a relatively small convenience sample. This might affect the generalizability of the findings
DuPlooy (2019) [63]	Online survey Australia	August to November 2016, online questionnaire	n = 1446 Anglo 528 (37%) Southern Asia 413 (29%) Confucian Asia 285 (20%) All other Europe 108 (8%) All other Countries 97 (7%)	Depression	1. People without internet access were not able to participate 2. Survey was only available in English and Chinese, people who were not able to speak these languages might have been prevented from taking part in the study 3. These are only associations and not able to make conclusions about causality
Brijnath (2020) [64]	Cross-sectional analysis of secondary data Australia	Data from the Australian 2015 National Health Survey	14,466 individuals ≥ 18 years who completed the K10	Psychological distress	1. Did not compare factors other than COB in analysis 2. The use of family members as interpreters may have biased responses around psychological distress 3. It also remains unclear what factors predispose Indian, Italian and Greek migrants to higher rates of distress than others
Rahman (2020) [65]	Cross-sectional, online survey, Australia	June 2020. Survey included residents in Australia, including patients, frontline health and other essential service workers, and community members	n = 587, the majority hailing from Victoria (88.2%). Mean age (± SD) of the participants was 41.3 (± 12.5) years and 61.8% were females	Psychological distress	The findings of this study were limited to people who could access online platforms to participate; hence, generalizability was limited to internet-literate people
Tabatabaei-Jafari (2021) [27]	Cross-sectional secondary data analysis New South Wales	45 and Up study	n = 228,039 Non-migrant 174,888, Migrant 53,151 Age ≥ 45 years	Psychological distress	1. This study sample consisted of middle- and older-aged adults, and thus the findings cannot be generalised to immigrants from other age groups 2. over-sampling of those in higher income groups, people aged over 80 years and those live in rural/regional
Demutska (2021) [31]	Online survey Victoria, Melbourne	September 2009 and April 2011	Russian speaking migrant 65, Russian speaking non-migrant 65, AA 63, Mean age 35 years	Anxiety, depression, stress	1. Small and convenient samples recruited through an online survey, limiting generalisability 2. No real risk-factor analysis done, only compared anxiety and depression by COB 3. No direct prevalence data reported
Bilal (2021) [66]	Cross-sectional study Northern Territory and Queensland, Darwin, and Brisbane	Period not found	Purposive sample of 170 adults (aged ≥ 18 years) Sub-Saharan African Migrants, Face-to-face interview. A migrant had to have lived in Australia for at least 2 years	Depression	Not including rural participants and those outside organized African community networks, to capture participants who are less socially connected with African communities

*ESC* English-speaking country of birth, *NESC* Non-English-speaking country of birth, *1G-NE* First generation non-English speaking background, *2G-NE* Second generation non-English speaking background, *AB* Australian-born host population (non-migrants), *AA* Anglo Australian, *COB* Country of birth

### Quality assessment of the papers

Twenty-seven studies had a minimum of three-stars in the selection domain, one-star in the comparability domain, and two-stars in the outcome/exposure domain, scored good quality. Only three studies (Maheshwari 2012, Meng 2014 and Bilal 2021) were found to be of poor quality, as they did not consider confounding factors during their analysis, leaving the outcome groups susceptible to non-compatibility.

### Prevalence of common mental health issues

People migrating from regions such as Southern and Eastern Europe, North Africa and The Middle East, North-East Asia and Sub-Saharan Africa to Australia reported greater rates of anxiety, depression and psychological distress than Australian-born non-migrants (Table 2).

**Table 2:** Prevalence of common mental health issues by place of birth according to the ‘Standard Australian Classification of Countries, 2016 (ABS)’

Region	Country	Issue	Prevalence among migrant	Prevalence among comparison group	Comparison group	Study
Oceania and Antarctica	–	Psychological distress	11.3%	12.4%	Australian-born host population	(Brijnath 2020) [64]
	New Zealand	Psychological distress	9.1%	12.4%	Australian-born host population	(Brijnath 2020)[64]
	Fiji	Anxiety and depression HSCL Mean (SD)	21.97 (3.21)	24.12 (3.31)	Australian-born host population	(Khawaja 2007) [49]
North-West Europe	–	Psychological distress	5.3%	12.4%	Australian-born host population	(Brijnath 2020)[64]
	United Kingdom	Psychological distress	8.5%	12.4%	Australian-born host population	(Brijnath 2020)[64]
	Germany	Psychological distress	9.8%	12.4%	Australian-born host population	(Brijnath 2020)[64]
	Switzerland	Psychological distress	4%	11%	Australian-born host population	(Feng 2013) [35]
	Denmark	Psychological distress	5%	11%	Australian-born host population	(Feng 2013) [35]
Southern and Eastern Europe	–	Psychological distress	18.2%	12.4%	Australian-born host population	(Brijnath 2020)[64]
	Italy	Psychological distress	18%	11%	Australian-born host population	(Feng 2013) [35]
		Psychological distress	20.7%	12.4%	Australian-born host population	(Brijnath 2020)[64]
		Depression	18%	vs 10%	Australian-born host population	(Stanaway 2010) [32]
	Malta	Psychological distress	17%	11%	Australian-born host population	(Feng 2013) [35]
	Greece	Depression, Mean (SD)	13.93 (9.62)	8.25 (9.11)	Australian-born host population	(Kiropoulos 2012) [53]
		Anxiety, Mean (SD):	45.08 (12.25)	33.85 (11.81)	Australian-born host population	(Kiropoulos 2012) [53]
		Psychological distress	20.4%	12.4%	Australian-born host population	(Brijnath 2020)[64]
		Psychological distress	20%	11%	Australian-born host population	(Feng 2013) [35]
		Depression	17.1%	4.1%	Australian-born host population	(Kiropoulos 2004) [29]
		Anxiety	43.1%	15.8%	Australian-born host population	(Kiropoulos 2004) [29]
	Russia	Depression, Mean (SD)	8.83 (7.85)	21.35 (11.64)	Australian-born host population	(Demutska 2021) [31]
		Anxiety, Mean (SD)	31.37 (8.83)	45.0 (11.51)	Australian-born host population	(Demutska 2021) [31]
		Stress, Mean (SD)	13.17 (5.88)	20.69 (8.29)	Australian-born host population	(Demutska 2021) [31]
	Poland	Psychological distress	18%	11%	Australian-born host population	(Feng 2013) [35]
North Africa and The Middle East	–	Psychological distress	21.9%	12.4%	Australian-born host population	(Brijnath 2020) [64]
	Lebanon	Psychological distress	33%	11%	Australian-born host population	(Feng 2013) [35]
		Anxiety and depression HSCL Mean (SD)	28.71 (2.25)	24.12 (3.31)	Australian-born host population	(Khawaja 2007) [49]
	Iran	Psychological distress	12.3%	19.2%	General students	(Nahidi 2018) [61]
South-East Asia	–	Psychological distress	5.8%	12.4%	Australian-born host population	(Brijnath 2020) [64]
	Vietnam	Anxiety disorders	3.1%	5.9%	Australian-born host population	(Steel 2009) [51]
		Anxiety-depression	7.0%	10.2%	Australian-born host population	(Liddell 2013) [54]
		Psychological distress	4.8%	12.4%	Australian-born host population	(Brijnath 2020) [64]
	Philippines	Depression	23%			(Thompson 2002) [46]
		Psychological distress	7.5%	12.4%	Australian-born host population	(Brijnath 2020)[64]
		Mean number of reported symptoms: Anxiety (At Baseline and at 5 years)	2.42 2.67	1.47 1.64	Australian-born host population	(Alati 2004) [33]
		Depression: At Baseline, at 5 years	1.35 1.35	0.810 0.77	Australian-born host population	(Alati 2004) [33]
North-East Asia	–	Psychological distress	8.6%	12.4%	Australian-born host population	(Brijnath 2020) [64]
	China	Depression	10%	3%	Australian-born host population	(Lin 2016) [30]
		Anxiety	6%	6%	Australian-born host population	(Lin 2016) [30]
		Anxiety	8%			(Dow 2018) [62]
		Depression	17%			(Dow 2018) [62]
		Psychological distress	6.1%	12.4%	Australian-born host population	(Brijnath 2020) [64]
		Psychological distress	12.9%	11%	Australian-born host population	(Feng 2013) [35]
		Psychological distress	18.8%			(Hu 2016) [58]
		Depression	30.8%			(Tang 2009) [52]
		Depression	19%			(Meng 2014) [55]
Southern and Central Asia	–	Psychological distress	11.9%	12.4%	Australian-born host population	(Brijnath 2020) [64]
	India	Psychological distress	10.8%	12.4%	Australian-born host population	(Brijnath 2020)[64]
	India	Psychological distress	15.4%			(Maheshwari 2012) [23]
Americas	–	Psychological distress	11.0%	12.4%	Australian-born host population	(Brijnath 2020)[64]
Sub-Saharan Africa	–	Depression	18.8%	9.3%	Australian-born host population	(Bilal 2021) [66]
		Psychological distress	11.2%	12.4%	Australian-born host population	(Brijnath 2020) [64]
Other Regions/ Types:	Asia	Psychological distress Mean (SD)	23.30 (2.20)	24.12 (3.31)	Australian-born host population	(Khawaja 2007) [49]
		Anxiety and depression	10–28%	20–35%	Australian-born host population	(Comino 2001) [48]
	Europe	Anxiety and depression HSCL Mean (SD)	27.88 (4.47)	24.12 (3.31)	Australian-born host population	(Khawaja 2007) [49]
		Anxiety and depression:	24–38%	20–35%	Australian-born host population	(Comino 2001) [48]
	Non-immigrant vs Immigrant	Psychological distress, mean (SD), Immigrant	14.06 (5.29)	13.91 (5.11)	Non-immigrant	(Tabatabaei-Jafari 2021) [27]
		Psychological distress, Immigrant	7%	6%	Non-immigrant	(Tabatabaei-Jafari 2021) [27]
		Psychological distress	61%	64%	Australian-born	(Rahman 2020) [65]
	CALD	Psychological distress	17.5%			(Ferdinand 2015) [56]
	English speaking or not	Depression: Male: ESB-FB,	9.2%	10.0%	Australian-born host population male	Straiton 2014) [21]
		Depression: Male: NESB-FB	19.7%	10.0%	Australian-born host population male	Straiton 2014) [21]
		Depression, Female ESB-FB	13.5%	18.1%	Australian-born host population female	Straiton 2014) [21]
		Depression, Female NESB-FB	20.2%	18.1%	Australian-born host population female	Straiton 2014) [21]
		Anxiety and depression: ESB	20–38%	20–35%	Australian-born host population	(Comino 2001) [48]
		Anxiety and depression: NESB	21–45%	20–35%	Australian-born host population	(Comino 2001) [48]
		Psychological distress: MESCs	2.0%	2.6%	Australian-born host population	(Sharma 2012) [34]
		Psychological distress: NESC	3.1%	2.6%	Australian-born host population	(Sharma 2012) [34]
		Affective disorder, ESC	5.9%	6.1%	Australian-born host population	(Liddell 2016) [59]
		Affective disorder, NESC	4.0%	6.1%	Australian-born host population	(Liddell 2016) [59]
		Anxiety disorders, ESC	11.0%	12.6%	Australian-born host population	(Liddell 2016) [59]
		Anxiety disorders, NESC	7.6%	12.6%	Australian-born host population	(Liddell 2016) [59]
		Common mental disorder, ESC	15.9%	19.2%	Australian-born host population	(Liddell 2016) [59]
		Common mental disorder, NESC	10.9%	19.2%	Australian-born host population	(Liddell 2016) [59]
	Asian and Western countries	Psychological distress at base, Mean (SD)	8.7 (6.1)	9.6 (6.0)	At 12-month f/up	(Chou 2007) [50]
	Africa	Anxiety and depression, HSCL Mean (SD)	21.40, (2.48)	24.12 (3.31)	Australian-born host population	(Khawaja 2007) [49]

Note: ESB, English speaking background; NESB, non-English speaking background; FB, Foreign born; ESC, English speaking country; NESC, non-English speaking country; MESCs, Main English-speaking countries

### Depression

Four studies reported a higher prevalence of depression in migrants compared to the non-migrants [29, 30, 32, 66]. Four other studies reported depression prevalence in migrants without comparing that with Australian-born [46, 52, 55, 62]. In one study, non-English speaking women reported the highest prevalence of depression (20.2%) [21], though the same study reported a higher prevalence of depression among non-migrants than migrants from English-speaking countries. Studies report variation in the prevalence of depression amongst people migrating from China (19.0%) [55], Africa (18.8%) [66], Italy (18%) [35], and Greece (17.1%) [29]. Two studies reported that depression was almost double in Italian migrants (18.0%) than non-migrants (10.0%) [32] and Sub-Saharan African migrants compared to Australia-born (18.8% vs. 9.3%) [66].

### Anxiety

The highest prevalence of anxiety disorders was reported by Kiropoulos and colleagues amongst people who migrated from Greece (43.1%), much higher than non-migrants (15.8%). Another study reported 6% of Chinese migrants had anxiety disorders with an equal proportion among Australian-born participants [30]. On the other hand, Liddell and colleagues reported the prevalence of anxiety disorders amongst migrants born in English-speaking countries (11.0%) and non-English speaking countries (7.6%) were lower than the Australian-born (12.6%) [67].

### Psychological distress

Eight studies reported the prevalence of psychological distress among migrants in Australia [23, 34, 35, 56, 58, 61, 64, 65]. The prevalence of psychological distress among migrants in Australia varies based on their country of origin, with notable differences between migrants from English-speaking and non-English-speaking backgrounds. Studies have reported lower levels of psychological distress among migrants from English-speaking regions such as Oceania (11.3%), North-west Europe (5.3%), and Americas (11%) compared to non-migrants (12.4%) [64]. Similarly, Sharma's study found that migrants from English-speaking countries reported lower psychological distress (2.0%) compared to non-migrants (2.6%) [34]. Feng’s study also reported a lower prevalence of psychological distress among migrants from Switzerland (4%) and Denmark (5%).

In contrast, migrants from non-English-speaking backgrounds, including South-East Asia (5.8%) [64], North Africa and the Middle East, where Lebanese migrants reported the highest psychological distress levels (33.0%), much higher than Australian-born (11%) [35], and Southern and Eastern Europe, with higher rates among migrants from Italy (20.7%) and Greece (20.4%), exhibited higher psychological distress than Australian-born individuals (12.4%) [64] (Table 2). Additionally, Maheswari et al. (2012) found a 15.4% prevalence of psychological distress among Indian-born migrants, and Hu and colleagues reported the prevalence of psychological distress among Chinese migrants in Australia was 18.8%, but they did not compare and report the prevalence among non-migrants.

### Other mental health issues

Few studies also reported stress and affective disorders and reported that these were lower among migrants than non-migrants [31, 67]. Two studies [51, 54] assessed the anxiety-depression altogether using the Composite International Diagnostic Interview (CIDI) 2.0 tool and both reported a lower prevalence among Vietnamese migrants than non-migrants (6.7–7% vs. 10.2–17.1%). Some studies did not report any prevalence data but compared the mean score of anxiety and/or depression among migrants and non-migrants (Table 2) using different tools (Appendix 2).

### Risk factors for mental health issues

**Fig. 2:**
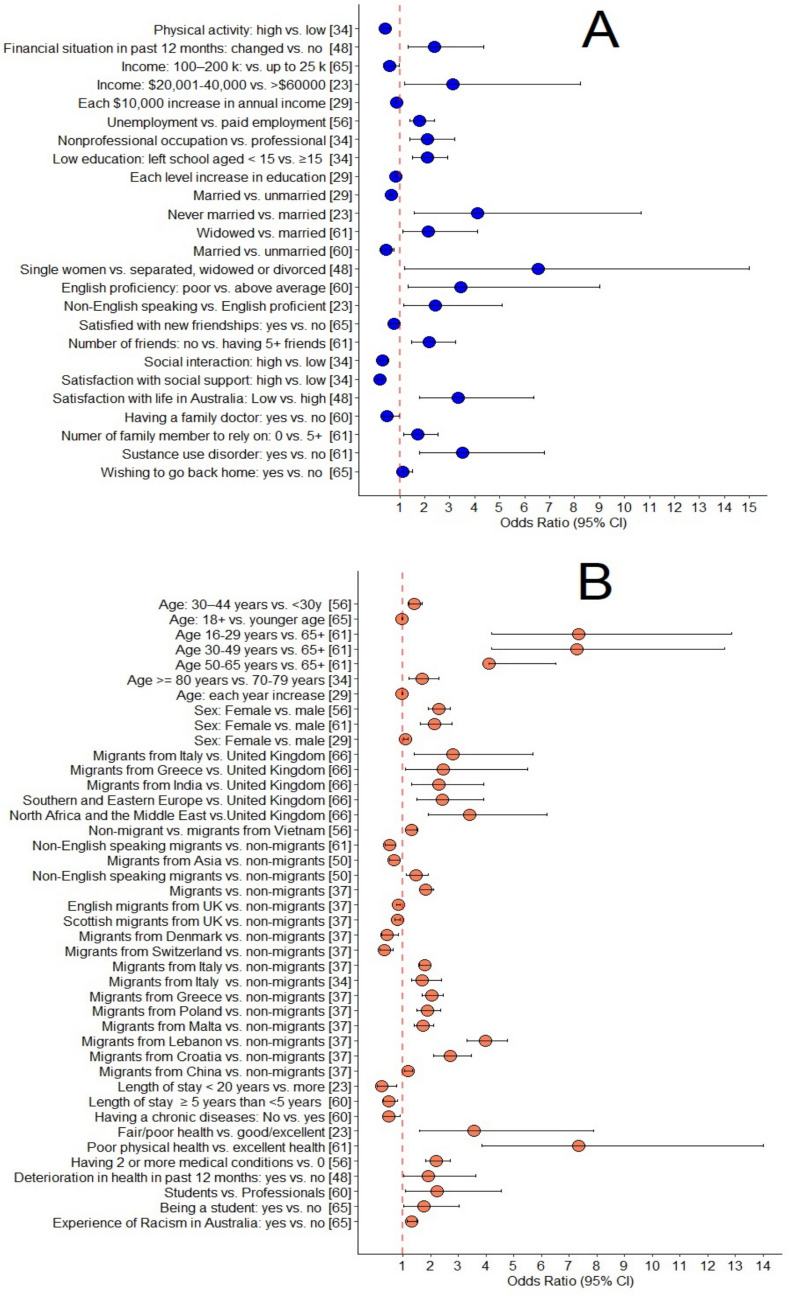
Modifiable (**A**) and non-modifiable (**B**) factors associated with anxiety disorders, depression and psychological distress among migrants in Australia: scoping review of papers published in 2000–2024

Significant factors linked to mental health issues among migrants in Australia as identified by several studies are divided into either ‘modifiable’ or ‘non-modifiable’ categories (Fig. 2). We constructed the Fig. 2 based on the odds ratios reported by the authors of the respective papers, where available.

#### Modifiable factors

One study reported that higher stress was positively associated with depression (p < 0.001) and anxiety disorders (p < 0.05) among migrants in Australia [29]. Another study reported that the greater the physical activity, the lesser the depression amongst migrants (Relative Risk 0.4, 95% CI 0.3–0.6) [32].

Three studies found racism and ethnic discrimination as risk factors. One study stated that migrants who experienced racism in Australia were more likely to report psychological distress than non-migrants (OR 1.31, 95% CI:1.14–1.50) [63]. Additionally, two studies reported a strong positive correlation (p < 0.01) between experiencing ethnic discrimination and depression [60, 66].

Many studies reported that migrants with lower levels of education and less proficiency in the English language were more likely to experience greater levels of depression than those with higher education (p < 0.01) [21, 49, 50, 55]. According to Stanaway et al., 2010, migrants who left school at age < 15 years were more likely to be depressed (RR 2.1, 95% CI:1.5–2.9) than those did not. Another study mentioned that the odds of having psychological distress was 16% lower for each level increase in education (no school, primary, high, diploma, university) [27]. Furthermore, Straiton et al., 2014 reported that migrants from non-English speaking countries had a higher risk of depression (OR 2.41, 95% CI:1.14–5.10) than non-migrants. Difficulty in speaking English was also found to be a significant predictor of psychological distress in two studies (p < 0.01) [49, 55] and the risk of having psychological distress was higher in those who had poorer English proficiency than average (AOR 3.44, 95%CI:1.31–9.02) [58].

The visa category was reported as another important factor predicting mental health status. Khawaja, 2007 reported that migrants with temporary visas were significantly more distressed than those with permanent visas/being Australian citizens (p < 0.01). According to Chou, 2007, skilled labour visa holders were more likely to report less psychological distress (p < 0.01) [50]. Two studies reported that student-visa holders were found to have a higher risk of psychological distress than Australian citizens (p < 0.05) [23, 50]. Depression rates among students were double compared to that among migrants working in professional fields, as reported by Hu et al. (2016) and du plooy et al. (2019); (AOR 2.22, 95% CI:1.08–4.54) [58], (OR 1.75, 95% CI:1.01–3.02) [63].

Several studies identified the financial situation as a significant predictor of mental health outcomes among migrants. Migrants who were economically inactive and experienced a poor financial situation were more likely to report distress (p < 0.01) as reported by Chou (2007) and depression (p < 0.001), as reported by Lin and colleagues [30]. According to Liddell et al. (2013), the risk of combined anxiety-depression was higher among unemployed than those in paid employment (OR 1.8, 95%CI:1.4–2.4) [54], similar to depression alone (p < 0.05) as found in one study [60]. Another study found that the relative risk of depression was found to be double for migrants whose source of income was from a non-professional occupation (RR 2.1, 95%CI:1.4–3.2) than from a professional occupation [32]. The level of income was another factor that predicted the likelihood of being psychologically distressed and depressed. One research found that migrants who earned up to AUD 25,000 annually reported more depression than migrants whose annual income was AUD 100,000–200,000 (OR = 0.57, 95% CI:0.33–0.96) [63]. Another research revealed that migrants with an annual income of AUD 20,000–40,000 reported more than three times the odds (OR 3.14, 95% CI:1.20–8.22) of depression than those earning > AUD 60,000 [21]. In their study, Tabatabaei-Jafari et al., (2021) mentioned that the odds of having psychological distress was 14% (95% CI 13–15%) lower for each AUD 10,000 increase in annual income. Besides, Thompson et al. (2002) reported that women who experienced a major deterioration in their financial status in the past 12 months were more than twice as likely to report psychological distress than women not experiencing a major change in their financial status in the past year (OR 2.38, 95% CI:1.31–4.37).

Being married was a protective factor against distress among migrants (AOR 0.43, 95% CI:0.24–0.77) as found by Hu and Wang (2016). Chou (2007) reported that migrants who were widowed and divorced or separated were more likely to report higher levels of distress (p < 0.01) than married. Similarly, psychological distress was 35% lower for married compared to unmarried as found by Tabatabaei-Jafari et al. (2021). Two other studies reported that unmarried status was associated with higher depression and anxiety disorders (p < 0.01) [29, 60]. As stated by Thompson et al. (2002), single women were more likely to have increased psychological distress (OR 6.54, 95%CI:1.18–35.3). Liddell et al. (2019) reported that widowed individuals were more likely to experience anxiety disorders compared to being married (OR 2.15, 95% CI:1.13–4.10).

Liddell et al. (2019) also mentioned that individuals who did not have any family members to rely on during stressful situations were more likely to experience anxiety disorders than those who had supportive family members (OR 1.70, 95% CI 1.16–2.52).

Some other factors associated with anxiety disorders, depression and distress amongst migrants were: having no friends (OR 2.18, 95%CI:1.47–3.22) according to Liddell et al. (2019), rare contact with friends (AOR 2.083, p < 0.001) as mentioned by Sharma (2012), poor satisfaction with life in Australia (OR 3.33, 95%CI:1.79–6.37) as stated by Thompson et al. (2002), willing to return to their home country (OR 1.13, 95% CI:1.13–1.52) as reported by du Plooy et al. (2019), and greater proportion of time spent alone each day (p < 0.05) as reported by Meng et al. (2014). On the other hand, high social interaction (RR 0.3, 95% 95% CI:0.2–0.5) found by Stanaway et al. (2010), being satisfied with new friendships (OR 0.78, 95% CI:0.63–0.97) according to du Plooy et al. (2019), having a family doctor in Australia (AOR 0.49, 95% CI:0.25–0.96) and awareness of available healthcare service providers (AOR 0.62, 95% CI:0.34–1.13) as found by Hu and Wang (2016) were associated with reduced risk of such mental health issues.

In summary, anxiety disorders, depression, and psychological distress amongst non-refugee migrants in Australia were associated with their level of education, English proficiency, visa type, financial stability, marital status, physical activity, experience of discrimination and racism, social isolation/loneliness, awareness of, and availability of social support and satisfaction with life in Australia.

#### Non-modifiable factors

Non-modifiable risk factors were defined as those outside of the individual's control (Fig. 2B). Of all non-modifiable factors, biological factors such as the age and sex of a migrant play an important role in predicting psychological distress, anxiety disorders, and depression. Age was differentially associated with mental health issues in different studies. Whereas some studies reported that the odds of having psychological distress and depression decreased with increasing age [27, 32], Hosseini et al. (2017) reported that being younger was associated with depression (p < 0.001). In their study, Liddell et al. (2019) found that anxiety disorder was more among migrants aged 16–29 years, 30–49 years, and 50–64 years in comparison to those aged ≥ 65 years (OR 7.34, 7.28 and 4.09 respectively). Another study reported that for every single-year increase in age, the likelihood of psychological distress increased by 2% (p < 0.05) [34]. The age at which s/he had migrated also plays a significant role: in the research by Meng et al. (2014), older age at the time of migration (p < 0.05) was associated with depression.

Although male migrants reported statistically significant (OR 2.22, 95%CI:1.37–3.58) increased risk of depression than Australian-born men, two other studies reported more (combined) anxiety-depression (OR 2.3, 95% CI:1.9–2.7) [54] and more anxiety disorders (OR 2.13, 95% CI:1.64–2.76) [67] in females than males. Also, Tabatabaei-Jafari et al. (2021) stated that the odds of having psychological distress was 8% greater for females than males (95% CI 1–17%).

How long one has been living in Australia is another predictor. The longer migrants lived in Australia, the less distress and depression were reported (p < 0.01) [50, 60]. In the study by Maheshwari and Steel (2012), residing in Australia for ≥ 5 years was less likely to be associated with mental health issues than residing < 5 years (AOR 0.49, 95% CI 0.30–0.81), similar findings reported by Hu and Wang (2016), p < 0.01). In a study, shorter living duration was also associated with depression (p < 0.05) [55]. In contrast, Straiton et al. (2014) reported that foreign-born men who had been in Australia for less than 20 years had a lower risk of depression (OR 0.23, 95% CI:0.07–0.78) than those living in Australia for over 20 years.

#### Some other factors

The overall self-reported physical health status, the presence of chronic illnesses, and the number of comorbid conditions experienced by migrants were associated with their mental health. There was a strong link between poor self-rated physical health status and mental health issues such as anxiety disorders and depression as mentioned by Lin et al. (2016) and Chou (2007) (p < 0.01). Straiton et al. (2014) mentioned that migrants who reported fair or poor physical health were three times more likely to report poor mental health compared to those who reported good or excellent physical health (OR 3.55, 95% CI:1.60–7.88). Similarly, Liddell et al. (2019) stated that migrants reporting poor physical health were more likely to experience anxiety disorders than those reporting excellent health (OR 7.35, 95% CI:3.86–14.01) and Thompson et al. (2002) reported that women whose physical health had deteriorated were more likely to experience psychological distress than women whose health had not changed in the past 12 months (OR 1.92, 95% CI:1.02–3.61).

Besides self-rated poor health status, a chronic health condition such as asthma, heart disease, rheumatism or arthritis, or diabetes can increase one’s vulnerability to psychological distress (p < 0.01) [34] and depression (p < 0.01) [50]. Bilal et al. (2021) found a strong relationship between diabetes and severe depression (p < 0.01). Conversely, it has also been found in one study that migrants without any chronic diseases had better mental health (AOR 0.48, 95% CI:0.26–0.89) [58].

Not only the presence of comorbidities but also the number of comorbidities is important in predicting the risk of mental health issues. One study reported that migrants with > 3 comorbid conditions had a three-fold (RR 2.9, 95% CI:2.1–3.9) increased risk of depression than those without comorbidity [32]. Another study found that migrants with two or more chronic medical conditions were more likely to report (combined) anxiety-depression (OR 2.2, 95% CI:1.8–2.7) than people without [54].

In brief, the migrant’s age, age of migration, gender, length of residence in Australia, physical health, presence of chronic diseases, and number of comorbid conditions- all were associated with their mental health status.

## Discussion

This review collated the findings from previous studies and found that non-refugee migrants in Australia reported a high burden of common mental health issues such as depression, anxiety disorders and psychological distress. The reported issues were based on participants’ self-reported symptoms in the anxiety disorders/depression domain and psychological distress. Few studies also found lower rates of problems in migrants in Australia than non-migrants, particularly where migration was from countries of origin from NW Europe, various parts of Asia, or New Zealand. Additionally, it revealed significant differences in the prevalence of these issues between migrants and their Australian-born non-migrant counterparts.

The prevalence of anxiety disorders, depression, and psychological distress varied among migrants from different countries at different times. Our scoping review identified that Northern Territory and South Australia are underrepresented in the migrant-related studies, which might indicate that the demographic and economic characteristics of these regions are less attractive to migrants to settle there than that of NSW and Victoria (e.g., About 80% of Bangladeshi-born migrants in Australia live in NSW and Victoria alone). Over half of the selected studies on migrants in Australia focused on depression, the majority reporting higher rates of depression among migrants compared to the Australian-born population with few exceptions. Migrants from non-English speaking countries like China, Africa, Italy, and Greece exhibited a high prevalence of depression. The highest prevalence of depression was found among non-English-speaking migrant women [21]. Some studies reported that migrants had a higher anxiety disorder (either percentage or mean score) than non-migrants, as high as 43.1% among migrants from Greece, and some studies reported a lower burden of anxiety disorders among migrants than non-migrants, as low as 7.0% among migrants from Vietnam. A few studies reported other common mental health issues such as stress and a high prevalence of psychological distress (up to 33%) amongst migrants in Australia [31, 35, 58, 64, 67]. It was hard to make a clear comparison among the findings reported in these studies due to methodological variations among different researchers and the application of different tools/instruments in different studies to estimate mental health issues.

Various factors can have a significant impact on mental health, including age at migration, duration of stay, country of origin, income, and experiences of discrimination [68]. Studies have indicated that migrants from ethnic and culturally distinct groups are more likely to face discrimination than the local majority population [61], and migrants from less developed countries are frequently seen more negatively than migrants from developed countries, regardless of their (individual) characteristics [69]. Discrimination was reported among foreign-born individuals living in other countries too [70]. Our review found evidence that discrimination and racism are predictors of mental health issues, and this needs further exploration and addressing in Australia. Among the migrants in Australia, other significant risk factors for mental health issues were being a student, holding a temporary visa, having diabetes or any other chronic health conditions, and low physical activity.

Country of birth (COB) is one vital predictor, because the likelihood of developing a mental health issue depends on the environment of the country where a migrant was born, their experience, growing up, learned language(s) and any situations that propelled them to leave their own country. English proficiency is another predictor that supports the findings of a systematic review of 25 studies globally where the pooled depression prevalence among migrants from non-English speaking countries was much higher (19.3%) than migrants from English-speaking countries (13.8%) [22]. The higher level of psychological issues reported by Chinese, Middle Eastern and African migrants in our scoping review can partly explained by a systematic review on language proficiency and mental disorders published by Montemitro et al. (2021) who stated, “Low language proficiency was generally found to negatively affect migrants’ mental health, being associated with increased prevalence and severity of psychiatric symptoms as well as mental disorders” (page no. 16) [71]. Another possible explanation could be the stigma around mental illness and poor help-seeking behaviour for mental health issues [72]. Furthermore, young age at migration and residing in Australia for a shorter duration (less than 5 years) were significant risk factors for depression compared to those who lived for a longer period in Australia, which may be a result of copping with the acculturation in a foreign country over time [73]. The prevalence of psychological distress and depression amongst migrants in Australia, in general, was found higher than that of the USA [13]. Some possible explanations could be that Australia has a more restrictive immigration policy, which may lead to increased stress and anxiety, particularly among those from non-English speaking backgrounds, as they navigate a new country and language. Also, Australia’s geographic isolation may exacerbate feelings of isolation and cultural dislocation for migrants. While migrants from Greece reported significantly lower levels of depression in Europe than non-migrants [18], we found that Greek migrants experienced a higher prevalence of depression, anxiety disorders, and high psychological distress than non-migrants in Australia as reported in multiple studies.

The present study found that poor annual income or deteriorating financial situation in the past year were strongly associated with migrants’ poor mental health. Studies outside Australia reported that a decline in household income was linked to an increased risk for incident mental illnesses, low levels of household income were linked to multiple lifetime mental diseases [74] and a significant association was found between income inequality and depression [75]. Therefore, there is a need for comprehensive interventions to prevent and treat mental issues among high-risk, low-income migrants.

Social isolation is recognized as a social determinant of mental health [76, 77]. Social isolation places an additional burden on society [78]. Loneliness has a detrimental effect on migrants’ resilience [79]. Our review provides evidence that social isolation, feeling homesick and lonely, living alone, separated, widowed, or divorced, rare contact with friends, dissatisfaction with friendship and limited social interaction—all are risk factors for depression among migrants. Attention needs to be paid to the larger social environment that may contribute to migrants’ feelings of social isolation [79].

The majority of studies in this scoping review focused on earlier waves of migrants to Australia such as Chinese, Vietnamese, Italian, Greek, Russian and European. Australia's migrant population influx has undergone a rapid transformation in recent times, with a notable change in the countries of origin. Specifically, there has been a substantial rise in the influx of South Asian migrants to Australia over the past decade. Australia is presently home to approximately 1,000,000 South-Asian immigrants comprising communities from the countries of India, Pakistan, Bangladesh and Sri Lanka [6]; the 2016 census recorded an increase of 48.3% from the 2011 Census. As of June 2021, 56,450 Bangladeshi-born people live in Australia [80]. Due to the limited number of published studies on the mental health issues of recent migrants, more research is needed to gain a better understanding of their challenges.

Contrary to the commonly held belief that migrants in Australia experience better health, a phenomenon known as the “healthy migrant effect”, where they tend to have better health outcomes than native-born Australians [20, 81], our findings do not align with this hypothesis concerning their mental health. Through a comprehensive exploration of mental health-related papers among non-refugee migrants in Australia, our research suggests that factors beyond the traditional ‘healthy migrant effect’ may significantly influence mental well-being in this population. Factors such as acculturation stress, discrimination, and socioeconomic disparities, may contribute to mental health challenges among non-refugee migrants in the Australian context- which require further research.

Another observation from our scoping review is that there is very limited information found about mental health issues amongst migrants in rural and regional Australia. Most studies were conducted in urban communities, therefore, there is a knowledge gap about the burden of mental illness among migrants in regional and rural Australia. In 2017, Silva et al. explored depression among rural people in New South Wales by interviewing General Practitioners (GPs). Long waiting times, insufficient patient rapport with referred professionals, cost of treatment, transportation, geographical location, stigma, and a lack of education about available mental health services were all identified by GPs as barriers to accessing mental health services in a rural setting [82]. Literature from the USA suggests that the prevalence of depression was significantly greater among rural people than in urban ones (6.1% vs 5.2%), perhaps due to differences in population characteristics [83]. We hypothesize that this can be true for rural and regional Australia too, which should be explored by future research.

Mental health issues were higher in regional areas compared with major cities in Australia [84]. Access to mental health services is much more limited in rural and regional areas than it is in major cities. There is a scarcity of health services of all types, particularly in specialized areas such as psychiatry in rural and regional Australia. Therefore, further research is required to understand the mental health issues among the migrants residing in rural and regional Australia compared with those in urban settings to tailor equitable mental health promotion and intervention strategies.

The role of English proficiency is crucial in evaluating mental health among migrant populations. Many standard mental health assessment tools, designed for English-speaking populations, often lack cultural sensitivity, making it challenging for migrants with limited English proficiency to understand and accurately respond as low English proficiency was reported in some studies under this scoping review. Therefore, attempts were made to use of culturally adapted tools and assessments in native languages [85, 86]. Cross-cultural adaptation of instruments is essential when the target population differs from the original population in terms of culture, country, or language in which the assessment tool was initially developed [87]. Therefore, mental health assessments for migrants should incorporate tools that account for both language barriers and cultural differences to ensure that their mental health needs are adequately addressed. Our review also noted that some researchers used different measurement instruments and found variations in prevalence rates [51, 62]. This was more profound when culturally specific tools were employed [51]. Hence, using culturally validated tools should be the highest priority for conducting mental health related studies.

There are certain limitations of the current scoping review to consider while interpreting the findings. Most of the studies were cross-sectional in nature, therefore causal relationships could not be established. In addition to that, the tools and methods used across the studies were heterogeneous, which limits comparisons among studies. Using refined keywords in the search strategy could potentially yield additional results, which could be another limitation. Due to the wide variations in methodologies among these studies, a meta-analysis was beyond the scope of this review.

## Conclusion

High rates of depression, anxiety disorders and psychological distress were observed among various non-refugee migrant groups in multicultural Australia. Although some studies reported a higher prevalence of such mental health issues among Australian-born non-migrants, more than half of the research showed a higher burden of these issues among migrants living in Australia. The high prevalence of common mental health issues among migrants highlights the need for targeted interventions to address this. Meanwhile, utilizing the existing community-based mental health initiatives, such as Community Mental Health Australia (https://cmha.org.au/), collaborating with the existing multicultural mental health resource centres, such as Victorian Transcultural Mental Health (https://vtmh.org.au/) and leveraging existing multicultural projects, such as Embrace Multicultural Mental Health initiative (https://embracementalhealth.org.au/) can be considered to advocate for the mental health needs of migrants. Mental health issues are associated with various risk factors, so identifying modifiable risk factors can help to develop prevention strategies. Research is scare on the mental health issues among migrants in rural and regional areas of Australia, specifically among newly emerging migrants. Hence, further research is needed to assess the burden of mental health issues among migrants in urban, regional, and rural areas and analyze potential predictors to tailor policies on healthcare services, healthcare access, health promotion, and intervention appropriate for their specific needs.

## Supplementary Information

Below is the link to the electronic supplementary material.

Supplementary file1 (DOCX 28 KB)

## Data Availability

The data that support the findings of this study are available on request from the corresponding author, PD following the institutional data-sharing policy.
