# Supplementary material for: Common Mental health issues among non-refugee migrants in Australia: a scoping review
Source: Soc Psychiatry Psychiatr Epidemiol. 2025 Feb 19;60(7):1515–40. doi: 10.1007/s00127-025-02850-2 (PMC12238152; doi:10.1007/s00127-025-02850-2)
Supplement: Supplementary file 1 — Supplementary file1 (DOCX 28 KB) [file 127_2025_2850_MOESM1_ESM.docx]

**APPENDIX 1**

Summary of quality assessment using the adapted Newcastle-Ottawa Scale.

| **Study** | **Selection** | | | | **Comparability** | **Outcome** | |  |
| --- | --- | --- | --- | --- | --- | --- | --- | --- |
|  | Representativeness of the sample | Sample size | Nonrespondents | Ascertainment of the exposure | Subjects in different outcome groups are comparable, outcome groups are comparable | Assessment of the outcome | Statistical test | Total |
| Rahman (2020) | * | * |  | ** | ** | * | * | 8 |
| Tang (2009) | * | * |  | ** | * | * | * | 7 |
| Ferdinand (2015) | * | * |  | ** | ** | * | * | 8 |
| Khawaja (2016) | * |  |  | ** | * | * | * | 6 |
| Nahidi (2018) | * | * | * | ** | ** | * | * | 9 |
| Dow (2018) | * |  |  | ** | * | * | * | 6 |
| Thompson (2002) | * | * | * | ** | * | * | * | 8 |
| Tabatabaei-Jafari (2021) | * | * | * | ** | * | * | * | 8 |
| Sharma (2012) |  | * |  | ** | * | * | * | 6 |
| Maheshwari (2012) |  | * | * | ** |  | ** | * | 7 |
| Lin (2016) |  | * |  | ** | * | ** | * | 7 |
| Liddell (2019) | * | * | * | ** | ** | * | * | 9 |
| Hosseini (2017) | * | * | * | * | * | * | * | 7 |
| Straiton (2014) | * | * | * | ** | ** | * | * | 9 |
| Steel (2009) | * | * | * | ** | * | * | * | 8 |
| Kiropoulos (2012) |  |  | * | ** | ** | * | * | 7 |
| Meng (2014) | * |  | * | ** |  | * | * | 6 |
| Hu (2016) |  | * | * | ** | ** | * | * | 8 |
| Stanaway (2010) | * | * | * | ** | ** | * | * | 9 |
| Kiropoulos (2004) | * | * | * | ** | ** | * | * | 9 |
| Khawaja (2007) | * |  |  | ** | * | * | * | 6 |
| Feng (2013) | * | * |  | ** | ** | * | * | 8 |
| duPlooy (2019) | * | * |  | ** | ** | * | * | 8 |
| Demutska (2021) |  |  |  | ** | ** | * | * | 6 |
| Liddell (2013) | * | * | * | ** | * | * | * | 8 |
| Comino (2001) |  | * | * | ** | ** | * | * | 8 |
| Chou (2007) | * | * | * | ** | ** | * | * | 9 |
| Brijnath (2020) | * | * | * | ** | ** | * | * | 9 |
| Bilal (2021) | * |  | * | ** |  | * | * | 6 |
| Alati (2004) |  |  | * | ** | ** | * | * | 7 |

*Good quality: 3 or 4 stars in the selection domain AND 1 or 2 stars in comparability domain AND 2 or 3 stars in outcome/exposure domain.Fair quality: 2 stars in selection domain AND 1 or 2 stars in comparability domain AND 2 or 3 stars in outcome/exposure domain. Poor quality: 0 or 1 star in selection domain OR 0 stars in comparability domain OR 0 or 1 stars in outcome/exposure domain.*

**NEWCASTLE - OTTAWA QUALITY ASSESSMENT SCALE (adapted)**

**Selection:** (Maximum 5 stars)

1) Representativeness of the sample:

a) Truly representative of the average in the target population. * (all subjects or random sampling)

b) Somewhat representative of the average in the target population. * (non-random sampling)

c) Selected group of users.

d) No description of the sampling strategy.

2) Sample size:

a) Justified and satisfactory. *

b) Not justified.

3) Non-respondents:

a) Comparability between respondents and non-respondents’ characteristics is established, and the response rate is satisfactory. *

b) The response rate is unsatisfactory, or the comparability between respondents and non-respondents is unsatisfactory.

c) No description of the response rate or the characteristics of the responders and the non-responders.

4) Ascertainment of the exposure (risk factor):

a) Validated measurement tool. **

b) Non-validated measurement tool, but the tool is available or described. *

c) No description of the measurement tool.

**Comparability:** (Maximum 2 stars)

1) The subjects in different outcome groups are comparable, based on the study design or analysis. Confounding factors are controlled.

a) The study controls for the most important factor (select one). *

b) The study control for any additional factor. *

**Outcome:** (Maximum 3 stars)

1) Assessment of the outcome:

a) Independent blind assessment. **

b) Record linkage. **

c) Self report. *

d) No description.

2) Statistical test:

a) The statistical test used to analyze the data is clearly described and appropriate, and the measurement of the association is presented, including  confidence intervals and the probability level (p value). *

b) The statistical test is not appropriate, not described or incomplete.

**APPENDIX 2**

Variation of measurement scales used by researchers in the papers to estimate the psychological distress, anxiety, and depression among migrants in Australia (2000-2022).

| **Scale used** | **Number of studies** | **Study reference (author date)** |
| --- | --- | --- |
| Kessler Psychological Distress Scale (K10) | 7 | Tabatabaei-Jafari 2021  Rahman 2020  Brijnath 2020  duPlooy 2019  Feng 2013  Sharma 2012  Maheshwari 2012 |
| Depression, Anxiety and Stress Scale (DASS) | 3 | Bilal 2021  Hosseini 2017  Khawaja 2016 |
| 12-item General Health Questionnaire (GHQ-12) | 2 | Chou 2007  Comino 2001 |
| Composite International Diagnostic Interview (CIDI) 2.0 | 2 | Liddell 2013  Steel 2009 |
| 28-item version of the General Health Questionnaire (GHQ-28) | 1 | Thompson 2002 |
| 4 separate scales used. GDS, Hospital anxiety and depression scale, De jong L scale, WHO quality of life questionnaire | 1 | Lin 2016 |
| Centre for Epidemiologic Studies Depression Scale, the State-Trait Anxiety Inventory, the Perceived Stress Scale | 1 | Demutska 2021 |
| Centre for Epidemiologic Studies for Depression Scale (CESD) | 1 | Straiton 2014 |
| Delusions-Symptoms- States-Inventory: State of Anxiety and Depression (DSSI/SAD) (Bedford and Foulds 1978). | 1 | Alati 2004 |
| Depressive illness was measured with the BDI-2. Anxiety was measured with the State-Trait Anxiety Inventory (state) | 1 | Kiropoulos 2004 |
| Depressive symptoms were measured with the BDI-2 | 1 | Kiropoulos 2012 |
| Geriatric Depression Scale [GDS] | 1 | Stanaway 2010 |
| Kessler 6-item scale | 1 | Hu 2016 |
| The Hopkins Symptom Checklist (HSCL) | 1 | Khawaja 2007 |
| Translated Chinese PHQ-9 questionnaire | 1 | Meng 2014 |
| World Mental Health Survey Initiative Composite International Diagnostic Interview (WHS-CIDI 3.0) | 1 | Liddell 2019 |
